# ASSISTANCE, PLANNING, AND UNMET NEEDS: FACTORS SHAPING OLDER ADULTS’ PET CARE BEHAVIORS IN THE UNITED STATES

**DOI:** 10.1093/geroni/igae098.3088

**Published:** 2024-12-31

**Authors:** Jessica Bibbo, Sarah Nicolay, Samantha Tuft

**Affiliations:** Benjamin Rose, Cleveland, Ohio, United States; Benjamin Rose, Cleveland, Ohio, United States; Benjamin Rose, Cleveland, Ohio, United States

## Abstract

Pets continue to be an important relationship for many people throughout life, while the abilities and resources available to provide for a pet change over the life course. This study surveyed people ages 50 and older on the assistance they had with pet care, whether they had gone without necessities to provide for their pet, and whether they had plans in place for their pet in case of emergencies or major life events. Participants (N=1,039) were enrolled in Foresight 50+, an omnibus survey representative of the US household population aged 50 or older. Descriptive statistics determined the proportion of adults who had experienced pet care behaviors along with specific types of assistance, necessity, and planning. A series of multivariate logistic regressions were conducted to identify associations between demographics and having assistance, going without, and planning. Most pet owners (72.4%) had some type of help with pet care, 18.8% had gone without at least one necessity to provide for their pet, and 23.7% had at least one plan in place for their pet. Larger household size was associated with having assistance (OR=1.96, p=.026). Lower income (OR=1.20, p=.024) and younger age (OR=1.06, p=.038) were associated with going without a necessity. Being female (OR=4.54, p=.003), smaller household size (OR=1.76, p=.036), and older age (OR=1.06, p=.010) were associated with having at least one plan in place. Available resources and personal characteristics significantly impact pet care behaviors. These results are a step toward better understanding how to support people and pets age in place.

